# The Prenatal Origin of Childhood Leukemia: Potential Applications for Epidemiology and Newborn Screening

**DOI:** 10.3389/fped.2021.639479

**Published:** 2021-04-23

**Authors:** Erin L. Marcotte, Logan G. Spector, Daniela P. Mendes-de-Almeida, Heather H. Nelson

**Affiliations:** ^1^Division of Epidemiology & Clinical Research, Department of Pediatrics, University of Minnesota, Minneapolis, MN, United States; ^2^Masonic Cancer Center, University of Minnesota, Minneapolis, MN, United States; ^3^Department of Hematology, Instituto Nacional de Infectologia Evandro Chagas, Fundação Oswaldo Cruz (FIOCRUZ), Rio de Janeiro, Brazil; ^4^Division of Molecular Carcinogenesis, Research Center, Instituto Nacional de Câncer (INCA), Rio de Janeiro, Brazil; ^5^Division of Epidemiology and Community Health, School of Public Health, University of Minnesota, Minneapolis, MN, United States

**Keywords:** leukemia, screening, newborns, translocation, epidemiology, childhood leukemia

## Abstract

Childhood leukemias are heterogeneous diseases with widely differing incident rates worldwide. As circulating tumors, childhood acute leukemias are uniquely accessible, and their natural history has been described in greater detail than for solid tumors. For several decades, it has been apparent that most cases of childhood acute lymphoblastic leukemia (ALL) and acute myeloid leukemia (AML) initiate *in utero*. Circumstantial evidence in support of this contention includes the young age of onset and high rate of concordance among identical twins. “Backtracking” of leukemic somatic mutations, particularly gene translocations, to cord blood and dried blood spots collected during the perinatal period has provided molecular proof of prenatal leukemogenesis. Detection of a patient's leukemia translocation in easily accessible birth samples, such as dried blood spots, is straightforward with the knowledge of their idiosyncratic breakpoints. However, to translate these findings into population-based screening and leukemia prevention requires novel methods able to detect translocations at all possible breakpoints when present in a low frequency of cells. Several studies have attempted to screen for leukemic translocations, mainly the common *ETV6-RUNX1* translocation, in cord blood samples from healthy children. Most studies have reported finding translocations in healthy children, but estimates of prevalence have varied widely and greatly exceed the incidence of leukemia, leading to concerns that technical artifact or contamination produced an artificially inflated estimate of translocation prevalence at birth. New generation techniques that capture the presence of these translocations at birth have the potential to vastly increase our understanding of the epidemiology of acute leukemias. For instance, if leukemic translocations are present at birth in a far higher proportion of children than eventually develop acute leukemia, what are the exposures and somatic molecular events that lead to disease? And could children with translocations present at birth be targeted for prevention of disease? These questions must be answered before large-scale newborn screening for leukemia can occur as a public health initiative. Here, we review the literature regarding backtracking of acute leukemias and the prevalence of leukemic translocations at birth. We further suggest an agenda for epidemiologic research using new tools for population screening of leukemic translocations.

## Introduction

Leukemia is the most common malignancy diagnosed in childhood. Acute lymphoblastic leukemia (ALL) comprises approximately 80% of all leukemia diagnoses among children age 0–19 years, and acute myeloid leukemia (AML) represents ~15–20%. ALL and AML demonstrate substantial differences in incidence patterns by age (1), race/ethnicity (2), and sex (3). Additionally, childhood forms of ALL and AML are distinct from those occurring in adulthood with respect to molecular (e.g., cytogenetic) features, demographic characteristics (e.g., incidence according to racial/ethnic group), risk factors, leukemogenic susceptibility associated with certain exposures, and prognosis. Advances in understanding of immunology and molecular/genetic features of the childhood acute leukemias along with laboratory improvements in immunophenotyping and cytogenetic characterization have led to the recognition of molecularly defined subtypes of ALL and AML, targeted therapeutics, and recognition of distinct prognostic groups (4). Notably, the past 30 years has also yielded insight into the natural history and etiology of acute leukemias, particularly with the discovery that many forms of childhood leukemia initiate *in utero* (5). This article reviews previous work identifying preleukemic clones present at birth, among both healthy children and those who later develop overt leukemia, and suggests a research agenda to capitalize on this knowledge.

### Epidemiology of Childhood Leukemia

#### Acute Lymphoblastic Leukemia

**Figure 1:**
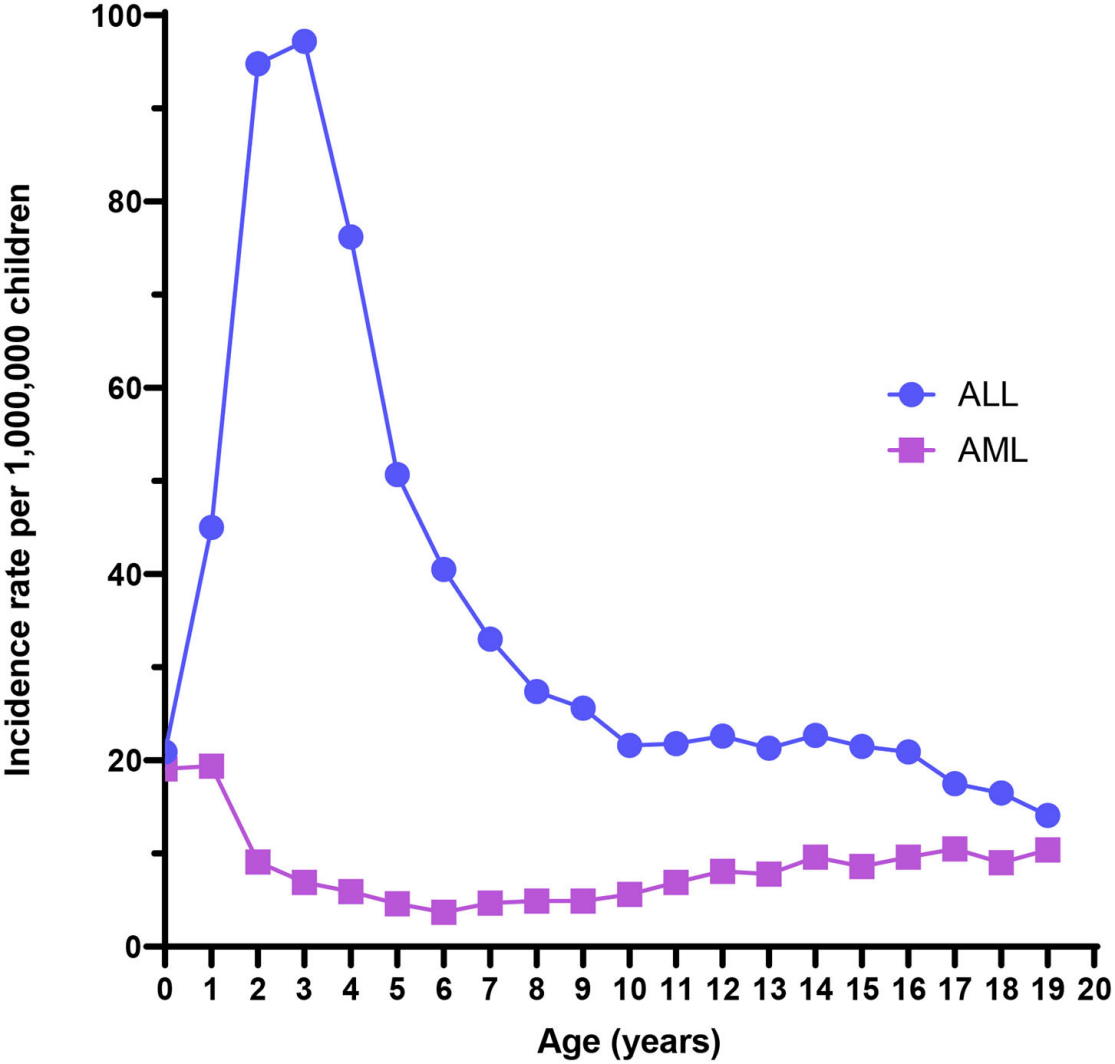
Acute lymphoblastic leukemia (ALL) and acute myeloid leukemia (AML) incidence rates from the US Surveillance, Epidemiology, and End Results program, 2000–2017.

ALL is the most common childhood cancer, with an overall incidence of 42 cases per million children. An early life peak in incidence is evident between 1 and 4 years of age, with rates nearing 100 cases per million (Figure 1). Males experience higher incidence rates compared with females at every age through 19 years (3), and both Hispanics and non-Hispanic whites have substantially higher risk of ALL than have African-American children (2). 5-year survival is high overall, approximately 87% for cases in the United States (4), but can be very low with unfavorable cytogenetics such as *KMT2A* rearrangements, *BCR*/*ABL* translocation, and intrachromosomal amplification of chromosome 21 (iAMP21) (6). ALL mortality does not completely describe the public health burden of the disease, as survivors later have markedly higher risks of cognitive deficits, obesity, debilitating chronic conditions, congestive heart failure, second neoplasms, and early mortality (7).

**Figure 2:**
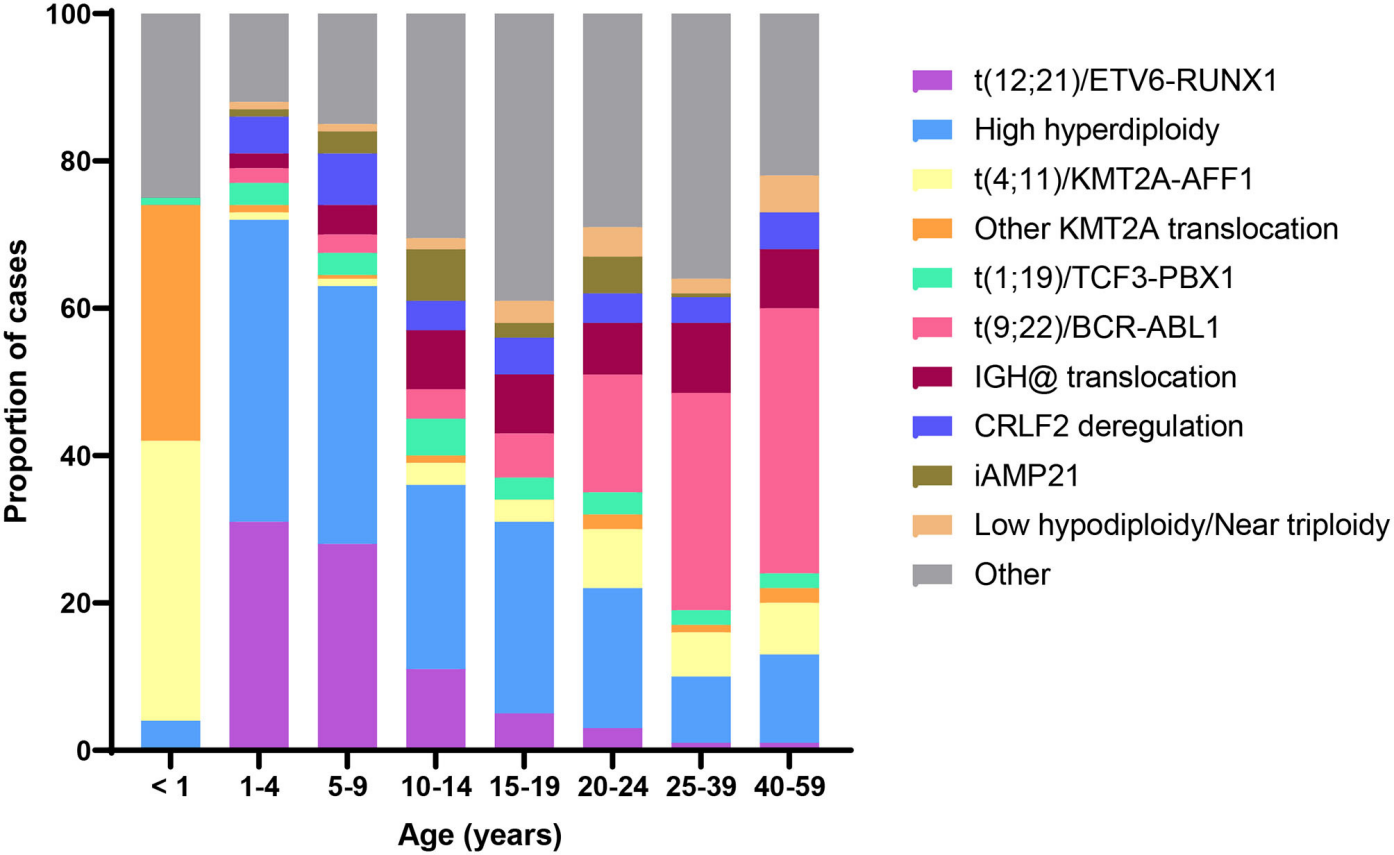
Distribution of B-cell acute lymphoblastic leukemia (ALL) cytogenetic subtypes by age at diagnosis. Data adapted from (6).

Acquired chromosomal and genetic abnormalities are a hallmark of ALL, and numerous structural and ploidy aberrations have been discovered (Figure 2). The two most frequent molecular events that dominate the early life peak include hyperdiploidy and the *t*_(12;21)_ translocation, which results in the *ETV6*/*RUNX1* gene fusion. Together, these somatic events are present in over 70% of ALL diagnosed between the ages of 1 and 4 years (6). By contrast, infant leukemia is largely driven by rearrangements in the *KMT2A* gene; the *t*_(4;11)_ translocation, which results in a *KMT2A*/*AFF1* gene fusion, is the most common and is present in ~2% of childhood ALL overall and 32% of infant leukemia (6). Figure 2 shows the distribution of cytogenetic and molecular profiles among ALL cases by age at diagnosis.

The etiology of ALL remains mostly obscure. Prenatal ionizing radiation is an accepted cause of ALL, but it is a rare exposure (8–11). No other environmental risk factors have emerged as definitively causal. For instance, meta-analyses of maternal alcohol use, paternal smoking, and moderate electromagnetic field exposure have reported odds ratios of 1.1, 1.11, and 1.25, respectively (12–16). Intrinsic risk factors have shown more consistent and stronger associations. Risk of ALL rises by about 8% for each 5-year increase in maternal age, while structural birth defects are associated with increases of about 50% (17, 18). ALL also independently rises as a linear function of birth weight (19–21). High-penetrance inherited syndromes underlie about 5% of cases, while common single nucleotide variants (SNVs) uncovered by genomewide association studies are estimated to account for 24% of the total variation in ALL risk (22). The highest magnitudes of association are seen with SNVs in *ARID5B, IKZF1, CEBPE, GATA3*, and *CDK2NA*; smaller associations are seen for SNVs in *COMMD3*/*BMI1* and *PIP4K2A*. Recent data from studies, which have examined ALL by cytogenetic or molecular subtypes, have found heterogeneity in associations with birth weight and SNVs. For instance, SNVs in *GATA3* and *PIP4K2A* have no association with *ETV6*/*RUNX1* ALL, whereas SNVs in *LHPP* and *ELK3* have modest effects on risk of *ETV6*/*RUNX1*. Polygenic risk scores have been developed for the ALL risk variants, and those in the top 1% of genetic risk have a 6.2-fold increased risk of developing ALL compared with those with the median level of genetic risk (23, 24). Additional susceptibility factors likely exist for ALL, but their discovery will require novel epidemiologic methods.

#### Acute Myeloid Leukemia

AML is a rare childhood malignancy that represents 15–20% of all leukemia diagnoses among children and has lower 5-year relative survival (67%) than childhood leukemia overall (85%) (25). The age-adjusted incidence rate of AML was 8.4 per million for children aged <19 years from 2000 to 2017 using data from the Surveillance, Epidemiology, and End Results program (Figure 1). The overall incidence rate is higher for males than females (3). Additionally, Asian/Pacific Islanders, American Indian/Alaska Natives, and Hispanics experience higher incidence rates than non-Hispanic whites or African-Americans. Finally, incidence is highest among infants compared with older age groups. The incidence has increased 0.7% per year from 2000 to 2017 (1). Few definitive risk factors for pediatric AML have emerged, and its etiology is largely unknown. Known risk factors for AML include race, exposure to ionizing radiation *in utero*, previous chemotherapy agents, bone marrow disorders such as myelodysplastic syndrome (MDS), and several genetic conditions, including Down syndrome (25, 26).

**Figure 3:**
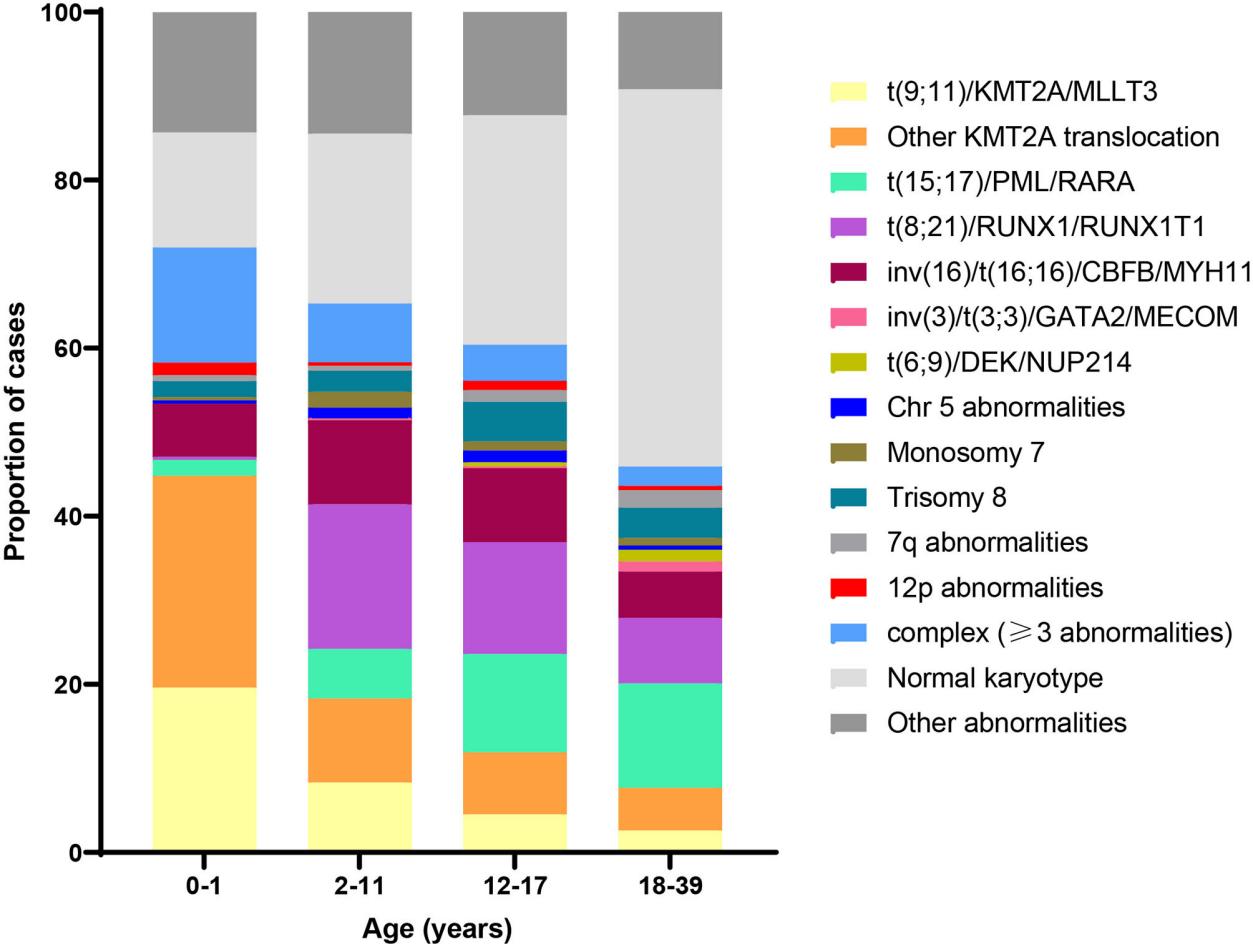
Distribution of acute myeloid leukemia (AML) cytogenetic subtypes by age at diagnosis. Data adapted from (30) and (29).

More than half of pediatric AML patients demonstrate an abnormal karyotype, with a subset exhibiting multiple abnormalities (27, 28). The most common include trisomy 8 and 21, monosomy 5 and 7, and various rearrangements such as *t*_(8;21)_, *t*_(15;17)_, and inv(16). Patients with a normal karyotype typically harbor recurrent mutations, frequently in *NRAS, KRAS, NPM1*, and *FLT3* (29). Recent evidence shows distinct age-related patterns in AML cytogenetics and mutations (29). For example, in contrast to older children, the most common abnormality in infants with AML is 11q23 translocation involving the *KMT2A* gene. Figure 3 shows the distribution of cytogenetic and molecular profiles among AML cases by age at diagnosis.

The diverse patterns of molecular characteristics observed in pediatric AML may result from specific exposome conditions that give rise to etiologically, clinically, and biologically distinct subsets of leukemia. The strongest evidence for this theory comes from studies of therapy-related AML, where different cytotoxic agents are associated with distinct molecular patterns (31, 32). Other studies suggest chemical exposure may be associated specifically with *RAS* mutation-positive AML (33, 34). Identifying the timing of molecular anomaly occurrence and the type of abnormalities may provide insight into AML etiology. Recognition that the 11q23 rearrangement is characteristic of exposure to topoisomerase II inhibitors in therapy-related AML has led to the investigation of maternal exposure to dietary and environmental inhibitors of topoisomerase II in relation to infant leukemia risk (35, 36). A prenatal origin also has been demonstrated for *t*_(8;21)_ in patients with childhood AML (37), which may be related to prenatal exposures (38). Although most cases of AML are sporadic, studies of familial clusters have identified case subgroups associated with germline mutations (39). The 2016 edition of the World Health Organization classification of hematopoietic neoplasms created a separate category for myeloid neoplasms with a germline predisposition that includes *CEBPA, RUNX1, ETV6, GATA2, DDX41*, and *ANKRD26* (40).

### *In utero* Initiation

#### Twin Studies

Anecdotal evidence of a prenatal origin of childhood leukemia first came in the form of case reports of twins and triplets concordant for leukemia. There have been over 70 twin pairs with concordant leukemia reported in the literature, the first of which was published in 1882 (41). The first molecular evidence for a prenatal origin of childhood leukemia arose from twin studies of *KMT2A*-rearranged and *ETV6*/*RUNX1* leukemia published in the 1990s (42–44). The first study published on this topic examined three pairs of identical infant twins with *KMT2A*-rearranged ALL and showed that the leukemia cells of each twin pair harbored *KMT2A* rearrangements that were identical within the pair but distinct from the other twin pairs as well as *KMT2A* rearrangements observed in eight infant ALL cases from singleton births. The first illustration of *in utero* origin of the *ETV6*/*RUNX1* translocation in twins appeared in the literature a few years later. This study demonstrated that a pair of monozygotic twins, diagnosed with ALL at ages 3 years 6 months and 4 years 10 months, respectively, harbored an identical *ETV6*/*RUNX1* fusion breakpoint. As the breakpoints for this translocation occur within a 14.7-kb region of *ETV6* (intron 1) and a 166-kb region of *RUNX1* (introns 1, 2, or 3), it would be nearly impossible for an identical breakpoint to occur by chance. The evidence provided by this study supports a model of leukemogenesis in which a single preleukemic clone arises *in utero* in one twin and is shared with the other twin via the shared placental blood. Since this time, several other publications have established identical translocations shared between identical twins.

#### Backtracking Studies

The observations from twin studies led to a series of “backtracking” studies providing further support for an *in utero* origin for preleukemic clones. In these studies, leukemia cells collected at the time of diagnosis were evaluated for cytogenetic and molecular abnormalities, including the exact patient-specific sequence of any gene translocations specific to the leukemia. Using this information, researchers examined each patient's DNA from dried blood spots (DBS) collected at birth for the purposes of newborn screening. Backtracking studies have revealed that, for each of the molecular events investigated, at least a portion of children diagnosed with leukemia harbored preleukemic cells at birth. The proportion of children whose unique leukemic event was detected in their newborn sample varies by subtype. Studies of hyperdiploid ALL have generally shown that a high proportion (83% to 100%) of patients harbored preleukemic cells at birth (45–48), although these studies were limited in the number of children screened (Table 1). By contrast, previous studies have reported a wide range of estimates for prevalence of other translocation events for *ETV6*/*RUNX1* (0–100%) (47–49, 54, 55, 62), *KMT2A*-rearranged (0% to 100%) (45, 47, 48, 53–57), *TCF3*/*PBX1* (13% to 100%) (47, 59), and *BCR*/*ABL1* (0% to 100%) (48, 60).

**Table 1:** Backtracking studies of childhood leukemia.

**Study, year**	**Disease**	**Age at diagnosis, range**	***N* screened**	***N* positive**	**% Positive**	**Population**	**Starting material**	**Screen**	**Detection method**
**ETV6/RUNX1 [*****t***_**(12;21)**_**]**									
Wiemels, 1999 (49)	B-ALL	2–5 years	11	8	73	Italy and United Kingdom	Newborn blood spot	DNA	LDI-PCR
Maia, 2001 (50)	B-ALL	21 months	2	2	100	nr	Newborn blood spot	DNA	PCR
Taub, 2002 (47)	B-ALL	2 years 11 months	1	1	100	United States (Michigan)	Newborn blood spot	DNA	PCR
Hjalgrim, 2002 (51)	B-ALL	2–6 years	9	3	33	Denmark	Newborn blood spot	DNA	nPCR
McHale, 2003 (52)	B-ALL	2–6 years	14	7	50	United States (California)	Newborn blood spot	DNA	LDI-PCR
Maia, 2004 (53)	B-ALL	5–11 years	7	3	43	United Kingdom, Italy, Germany, United States, New Zealand	Newborn blood spot	DNA	LDI-PCR
Burjanivova, 2006 (54)	B-ALL	3–5 years	3	1	33	Czech	Newborn blood spot	DNA	PCR
Gruhn, 2008 (48)	B-ALL	1–6 years	6	3	50	Germany	Newborn blood spot	DNA	snPCR
Taylan, 2019 (55)	ALL	1–4 years	4	0	0	Sweden	Newborn blood spot	DNA	dPCR
**KMT2A/AFF1 [*****t***_**(4;11)**_**]**									
Gale, 1997 (56)	B-ALL	5 months to 2 years	3	3	100	United Kingdom	Newborn blood spot	DNA	PCR
Fasching, 2000 (57)	B-ALL	6 months to 2 years	2	2	100	Austria	Newborn blood spot	DNA	PCR
Yagi, 2000 (45)	B-ALL	1–2 months	2	2	100	nr	Newborn blood spot	DNA	nPCR
Taub, 2002 (47)	B-ALL	10 years	1	1	100	United States (Michigan)	Newborn blood spot	DNA	PCR
Maia, 2004 (53)	ALL	5–8 years	3	1	33	United Kingdom, Italy, Germany, United States, New Zealand	Newborn blood spot	DNA	LDI-PCR
Gruhn, 2008 (48)	B-ALL	2 years 5 months	1	0	0	Germany	Newborn blood spot	DNA	snPCR
**Other KMT2A-r**									
Maia, 2004 (53)	ALL	5 years	1	0	0	United Kingdom, Italy, Germany, United States, New Zealand	Newborn blood spot	DNA	LDI-PCR
Maia, 2004 (53)	AML	3 years	1	0	0	United Kingdom, Italy, Germany, United States, New Zealand	Newborn blood spot	DNA	LDI-PCR
Burjanivova, 2006 (54)	AML	1–5 years	3	0	0	Czech	Newborn blood spot	DNA	PCR
Taylan, 2019 (55)	ALL	1 month	1	1	100	Sweden	Newborn blood spot	DNA	dPCR
**Hyperdiploidy**									
Maia, 2004 (58)	ALL	1–10 years	11	1	9	nr	Cord blood	DNA	Taqman
Yagi, 2000 (45)	B-ALL	2 years	1	1	100	nr	Newborn blood spot	DNA	nPCR
Taub, 2002 (47)	B-ALL	2–9 years	5	5	100	United States (Michigan)	Newborn blood spot	DNA	PCR
Gruhn, 2008 (48)	ALL	2–14 years	6	5	83	Germany	Newborn blood spot	DNA	snPCR
Panzer-Grumayer, 2002 (46)	B-ALL	2 years	1	1	100	Austria	Newborn blood spot	DNA	nPCR
**RUNX1/RUNX1T1 [*****t***_**(8;21)**_**]**									
Burjanivova, 2006 (54)	AML	9–14 years	2	0	0	Czech	Newborn blood spot	DNA	PCR
Wiemels, 2002 (37)	AML	3–12 years	10	5	50	United Kingdom and United States (California)	Newborn blood spot	DNA	PCR
**TCF3/PBX1 [*****t***_**(1;19)**_**]**									
Taub, 2002 (47)	B-ALL	13 years	1	1	100	United States (Michigan)	Newborn blood spot	DNA	PCR
Wiemels, 2002 (59)	B-ALL	1–12 years	15	2	13	United States (California)	Newborn blood spot	DNA	LDI-PCR
**BCR/ABL [*****t***_**(9;22)**_**]**									
Gruhn, 2008 (48)	B-ALL	1 year	2	0	0	Germany	Newborn blood spot	DNA	snPCR
Cazzaniga, 2011 (60)	ALL	5 years	1	1	100	United Kingdom	Newborn blood spot	DNA	PCR
**PML/RARA [*****t***_**(15;17)**_**]**									
McHale, 2003 (61)	AML	10 years	2	1	50	United States (California)	Newborn blood spot	DNA	LDI-PCR
Burjanivova, 2006 (54)	AML	7–13 years	4	0	0	Czech	Newborn blood spot	DNA	PCR
**Other**									
Fasching, 2000 (57)	B-ALL	4 years 8 months	1	1	100	Austria	Newborn blood spot	DNA	PCR
Fasching, 2000 (57)	T-ALL	2 years	2	2	100	Austria	Newborn blood spot	DNA	PCR
Yagi, 2000 (45)	B-ALL	1–9 years	4	1	25	nr	Newborn blood spot	DNA	nPCR
Taub, 2002 (47)	B-ALL	18 months to 9 years	9	4	44	United States (Michigan)	Newborn blood spot	DNA	PCR
McHale, 2003 (61)	AML	9–14 years	2	1	50	United States (California)	Newborn blood spot	DNA	LDI-PCR
Burjanivova, 2006 (54)	B-ALL	1–4 years	9	2	22	Czech	Newborn blood spot	DNA	PCR
Gruhn, 2008 (48)	B-ALL	1–13 years	17	11	65	German	Newborn blood spot	DNA	snPCR
Burjanivova, 2006 (54)	AML	2–8 years	4	0	0	Czech	Newborn blood spot	DNA	PCR
Taylan, 2019 (55)	ALL	3 years	2	0	0	Sweden	Newborn blood spot	DNA	dPCR

*nr, not reported; dPCR, digital PCR; nPCR, nested PCR; snPCR, semi-nested PCR; ALL, acute lymphoblastic leukemia; AML, acute myeloid leukemia*.

## Previous Screening Studies of Healthy Newborns

There is substantial evidence that at least some of the common leukemia translocations are not necessarily deterministic for future development of leukemia and are present at varying prevalence in umbilical cord blood (UCB) of healthy newborns (Table 2) (62–70, 73, 74). The *ETV6*/*RUNX1* translocation is by far the most widely studied preleukemic event among an unselected pool of healthy newborns. To our knowledge, the *TCF3*/*PBX1, KMT2A*/*AFF1, BCR*/*ABL*, and *RUNX1*/*RUNX1T1* translocations are the only other leukemia translocation events to be examined among a sample of unselected newborns (Table 2).

**Table 2:** Studies of healthy newborns that screened for leukemia translocations.

**Study, year**	***N* screened**	***N* positive**	**% Positive**	**Population**	**Starting material**	**Age group**	**Screen**	**Detection method**	**Confirmation method**	**Estimated cell frequency**
**ETV6/RUNX1 [*****t***_**(12;21)**_**]**										
Eguchi-Ishimae, 2001 (62)	67	1	1.5	Japanese	Cord blood	Newborns	RNA	nRT-PCR		
Mori, 2002 (63)	567	6	1.1	British	Frozen cord blood	Newborns	RNA	nRT-PCR, qRT-PCR	FISH	10^−3^ to 10^−4^
Lausten-Thomsen, 2010 (64)	256	0	0.0	Danish	Fresh cord blood	Premature newborns	RNA	qRT-PCR		
Lausten-Thomsen, 2011 (65)	1,417	0	0.0	Danish	Fresh cord blood	Newborns	RNA	qRT-PCR	qRT-PCR	
Zuna, 2011 (66)	253	5	2.0	Czech	Cord blood	Newborns	RNA	qRT-PCR	FISH	
Olsen, 2012 (67)	1,258	3	0.2	Danish	Fresh cord blood	Newborns	RNA	qRT-PCR	Sanger	< 10^−4^
Ornelles, 2015 (68)	210	5	2.4	United States	Fresh cord blood	Newborns	RNA	nRT-PCR	Sanger	≤ 10^−5^
Kosik, 2017 (including Skorvaga et al.) (69)	500	nr	2.4	Slovak	Cord blood	Newborns	RNA	qRT-PCR	qRT-PCR and Sanger	≤ 10^−5^
Schafer, 2018 (70)	1,000	50	5.0	Danish	Frozen cord blood	Newborns	DNA	GIPFEL	Sanger	10^−2^ to 10^−5^^*^
**KMT2A/AFF1 [*****t***_**(4;11)**_**]**										
Zuna, 2011 (66)	103	0	0.0	Czech	Cord blood	Newborns	RNA	RT-PCR		
Kim-Rouille, 1999 (71)	60	0	0.0	nr	Cord blood	Newborns	RNA	RT-PCR	Sanger	
Kosik, 2017 (including Skorvaga et al.) (69)	500	nr	0.8	Slovak	Frozen cord blood	Newborns	RNA	qRT-PCR	qRT-PCR and Sanger	
BCR/ABL [*t*_(9;22)_]
Zuna, 2011 (66)	103	0	0.0	Czech	Cord blood	Newborns	RNA	RT-PCR		
Kosik, 2017 (including Skorvaga et al.) (69)	500	nr	5.0	Slovak	Frozen cord blood	Newborns	RNA	qRT-PCR	qRT-PCR and Sanger	
**RUNX1/RUNX1T1 [*****t***_**(8;21)**_**]**										
Mori, 2002 (63)	496	1	0.2	British	Frozen cord blood	Newborns	RNA	nRT-PCR, qRT-PCR	FISH	
**TCF3/PBX1 [*****t***_**(1;19)**_**]**										
Hein, 2019 (72)	340	2	0.6	Danish	Frozen cord blood	Newborns	DNA	GIPFEL	RT-PCR and Sanger	

*nr, not reported; FISH, fluorescence in situ hybridization*.

* *Among sorted CD19+ cells*.

### Translocation Prevalence Estimates and Variation

**Figure 4:**
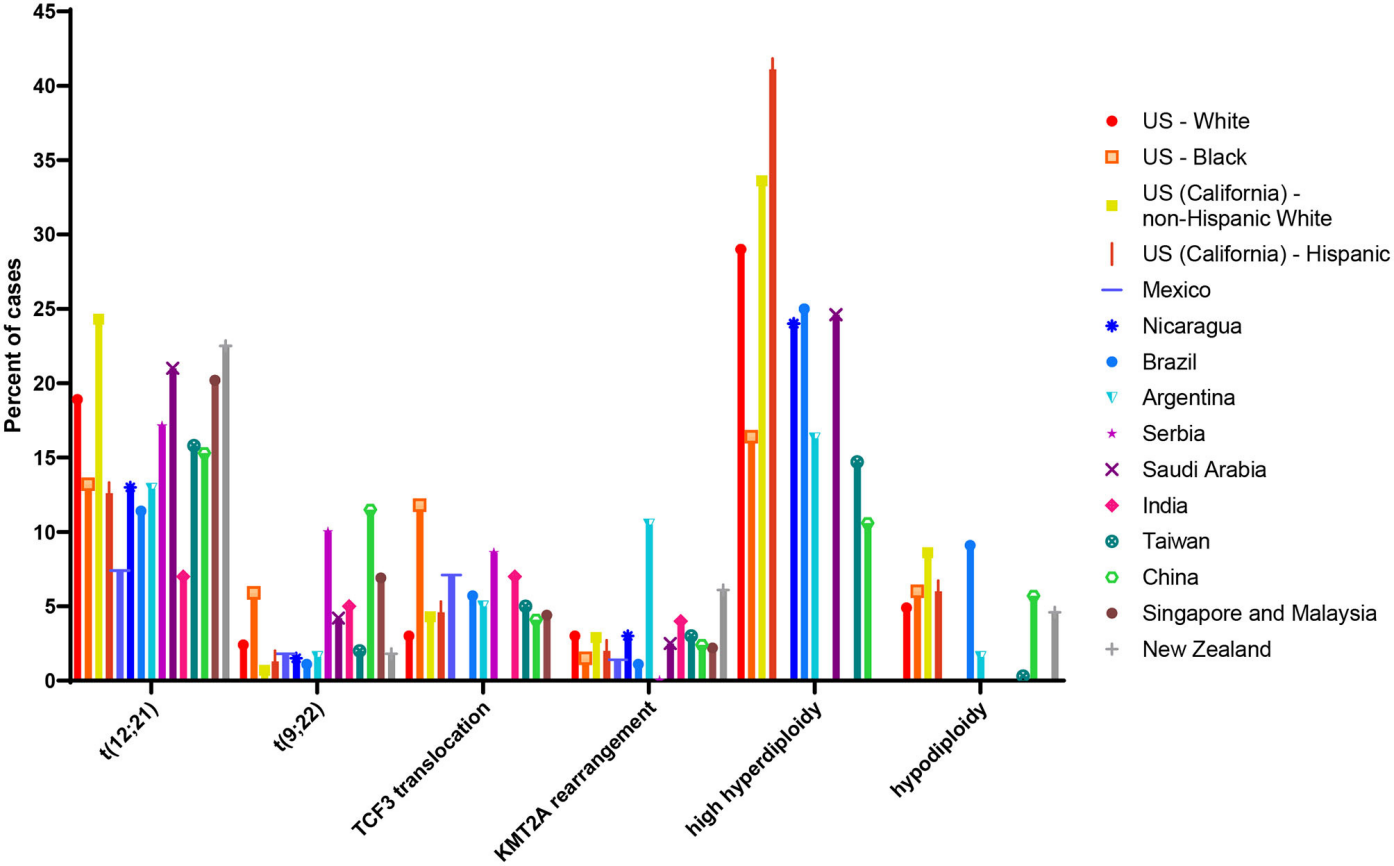
Proportion of common cytogenetic and molecular abnormalities among acute lymphoblastic leukemia (ALL) cases of different racial and ethnic groups or nations. Estimate of cytogenetic distribution among children diagnosed in the following countries: US white and black children and adolescents (age range not reported) taken from (79); US (California) Hispanic and non-Hispanic white children (age 0–14 years) taken from (76); Mexico (age 0–18 years) taken from (80); Nicaragua (age 0–16 years) taken from (81); Brazil (age 0–17 years) taken from (82); Argentina (age 0–16 years) taken from (83); Serbia (age 0–16 years) taken from (84); Saudi Arabia (age 0–14 years) taken from (85); India (age 0–20 years) taken from (86); Taiwan (age 0–18 years) taken from (77); China (age 0–18 years) taken from (87); Singapore and Malaysia (age 0–16 years) taken from (88); New Zealand (age 0–14 years) taken from (78). Taiwanese children (age 0–18 years) taken from (77); estimate of cytogenetic distribution among Chinese children (age 0–18 years) taken from (87).

Estimates of the *ETV6*/*RUNX1* translocation prevalence among all newborns at birth have varied widely (75), from 0.01% to 5% (65, 70). The sources of this variation may be due to the translocation detection methods and materials used (discussed below). Differences in populations tested may also lead to variation in estimated population frequency. Indeed, previous literature on the distribution of cytogenetic abnormalities among ALL patients of varying racial and ethnic groups has suggested that *ETV6*/*RUNX1* leukemia is less common in Hispanics and East Asian populations compared with populations of European ancestry (76–78). Figure 4 shows estimates of cytogenetic abnormality frequency by country, from publications that reported proportions of at least four of the most common abnormalities (76–88). To our knowledge, two previous screening studies have focused on non-white populations. Eguchi-Ishimae et al. screened cord blood samples from 67 Japanese newborns and reported one (1.5%) as positive for the *ETV6*/*RUNX1* translocation (62). Ornelles et al. conducted the only study with a majority African-American and Hispanic population. Of the 210 cord blood samples screened, 148 were from African-American newborn and 55 were from Hispanic newborns (68). Three (2.0%) samples from African-American children were positive for the *ETV6*/*RUNX1* translocation, while two (3.6%) samples from Hispanic children were positive.

**Figure 5:**
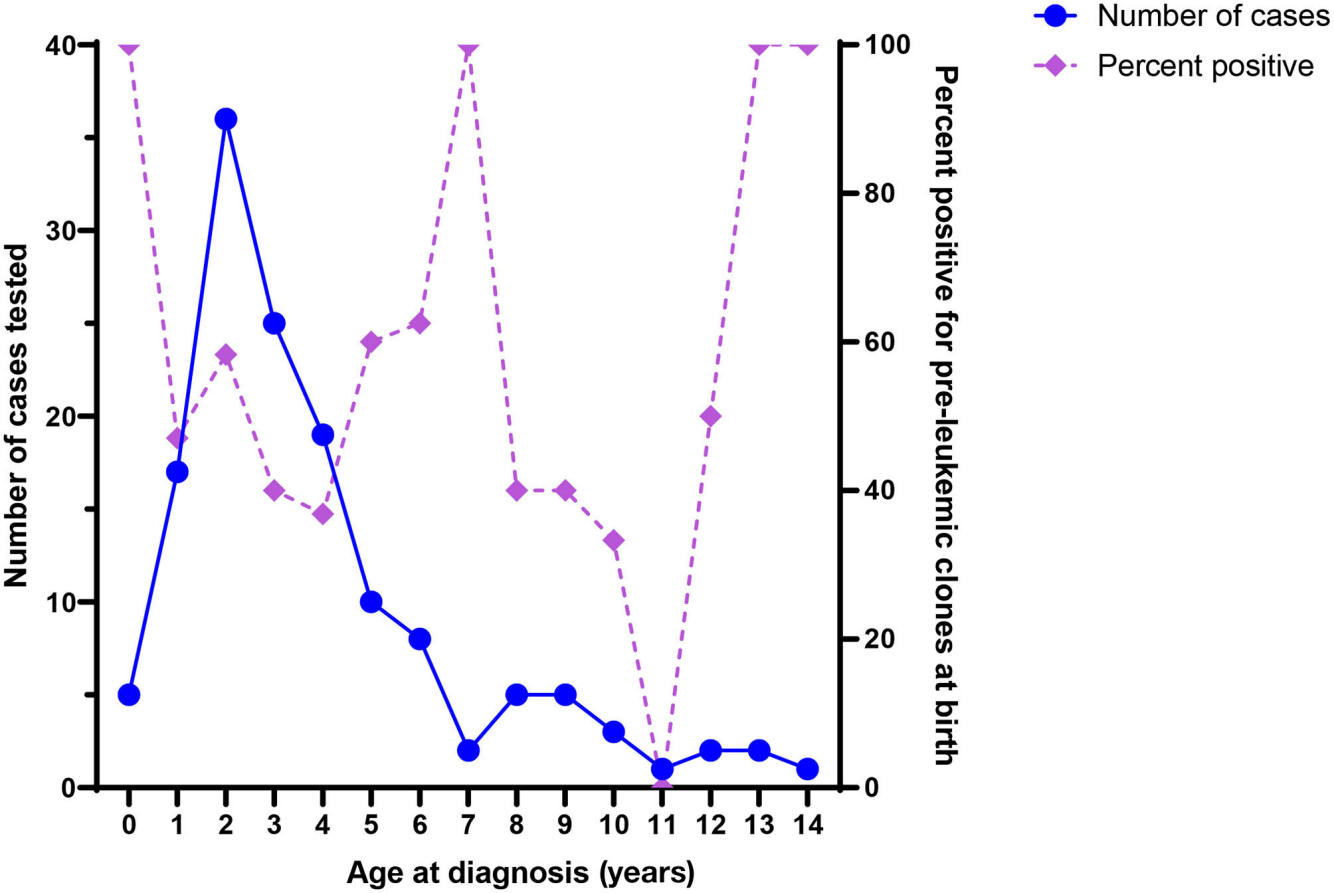
Number of acute lymphoblastic leukemia (ALL) cases tested in backtracking studies, by age at diagnosis (left axis), and the proportion of cases positive for preleukemic clones at birth, by age at diagnosis (right axis).

It is notable that ALL-associated translocations have been reported more commonly than those associated with AML, in both backtracking (Table 1) and screening (Table 2) studies. There are several possible explanations for this phenomenon. The first is that the relative frequency of childhood ALL, and *ETV6*/*RUNX1* ALL in particular, compared with childhood AML has led to more investigation of this leukemia subtype. In the published literature presented in Table 1, a total of 158 unique ALL patients have been examined in backtracking studies, of which 76 [48% (95% confidence interval, calculated by the Wilson score interval (89): 40–56%)] were positive for preleukemic clones at birth. By contrast, a total of only 28 AML patients have been examined in backtracking studies, of which seven [25% (95% confidence interval, calculated by the Wilson score interval (89): 13–43%)] were positive for preleukemic clones at birth. The apparent lower birth prevalence of preleukemic clones among children later diagnosed with AML is statistically indistinguishable from that of children later diagnosed with ALL and may simply be due to the small number of patients examined. Additionally, the presumption of an *in utero* origin is the strongest for cancers occurring near birth and diminishes with age. As AML is less skewed toward a younger age at onset than ALL (Figure 1), the observed detection of clones in birth samples may reflect true differences in frequency of *in utero* initiation. We examined whether the available data support the hypothesis that the age-specific incidence curves of ALL and AML indicate a likely lower frequency of *in utero* initiation of AML. We abstracted age at diagnosis and presence of preleukemic clones at birth for all 158 ALL cases reported in publications in Table 1, with the exception of 15 t(1;19)+ B-ALL cases in Wiemels et al. (59) because it was not possible to determine which of the patients presented in the publication were included in the Guthrie card analysis. Figure 5 shows number of ALL cases tested by age at diagnosis and proportion of those with detectable preleukemic clones at birth. We conducted a simple logistic regression to determine whether age at diagnosis (in months) was associated with odds of preleukemic clone presence at birth and observed no relationship (odds ratio = 0.99; 95% confidence interval: 0.98–1.01). Another explanation is that AML-associated translocations are more difficult to detect than the common ALL-associated translocations. Indeed, while the *ETV6*/*RUNX1* translocation occurs in predictable regions of the genes involved—the breakpoints occur within a 14.7-kb region of *ETV6* (intron 1) and a 166-kb region of *RUNX1* (intron 1, 2, or 3) (90)—*KMT2A* rearrangements include more than 90 known translocation partner genes, 35 of which are known to occur recurrently (91). Additionally, the most common of these, *KMT2A*/*AFF1* translocation, occurs more frequently among infant leukemia patients with ALL rather than AML (91, 92). The other common recurrent AML-associated translocations, including *RUNX1*/*RUNX1T1, PML*/*RARA*, and inv(16), have been investigated in few backtracking studies (Table 1); and only one of these, *RUNX1*/*RUNX1T1*, has been investigated in a screening study published in 2002 (Table 2). Thus, more research is needed on the *in utero* initiation of AML to determine its frequency, both in children who later develop AML and in the general population of healthy newborns.

### Methods and Material Used

Uncertainty around these prevalence estimates, in both cases and healthy controls, relates to the ability to detect a rare event in a vast excess of normal cells. Detection of rare translocations can be achieved using DNA if the exact translocation is known, as is the case for backtracking of known ALL cases (37, 41, 49, 52, 93). However, this approach is not feasible for screening unless the breakpoint region is small, which is not the case for *ETV6*/*RUNX1*; the breakpoints for this translocation occur within a 14.7-kb region of *ETV6* (intron 1) and a 166-kb region of *RUNX1* (intron 1, 2, or 3). Therefore, RNA approaches have typically been employed for the detection of the *ETV6*/*RUNX1* translocation product in birth samples. The prior research has relied heavily on the use of nested reverse transcriptase PCR or quantitative RT-PCR to detect the low copy signal, and this is problematic, as RNA-based PCR methods are prone to contamination and false-positive results. To limit the contribution of false positives, most prior studies used at least one validation method (Table 2), but questions on the variation in prevalence between studies remain. RNA methods for detection of the *ETV6*/*RUNX1* translocation product produce the same transcript for each breakpoint, which diminishes the ability to identify contaminants. Additionally, false-negative results are possible with low-quality input material.

Previous studies have relied on UCB or flow-sorted B cells from the cord blood. There are also differences in processing and storage of UCB in prior studies, some of which used fresh cord blood processed within 24 h of birth, while others used frozen UCB. Both of these methods are problematic for larger, population-based studies, as UCB is not routinely collected after birth and requires substantial resources for collection, processing, and storage. Thus, UCB-based methods are not scalable for very large population-based studies or implementation in newborn screening.

Development of highly sensitive DNA-based methods for translocation detection is a desirable step forward for this work. DNA is far more stable than RNA, and DNA-based translocation detection would facilitate identification of contamination since patient-specific amplification products can be compared with respect to size and exact sequence. Indeed, given these advantages, DNA-based methods have been utilized clinically in minimum residual disease monitoring (94). Recently, a DNA-based method for detection of translocation events at birth has been described, GIPFEL, and in among 1,000 unselected newborns has produced the highest estimate for birth prevalence of the *ETV6*/*RUNX1* translocation (50/1,000; 5%) (70). While a DNA-based method has many advantages over RNA-based methods, the literature published using this method also required UCB processed within 24 h of birth as the input material to enable flow-sorting enrichment of CD19+ B cells (95). Currently, it is not feasible for use in population-wide screening; however, if the method is optimized to use whole blood with lower requirements for input material, it may 1 day be possible to deploy in newborn DBS.

## Future Research Agenda

### Advancing Novel Translocation Screening Methods

#### Utility of Newborn Dried Blood Spots

Newborn screening is a mandatory public health program in the United States and most high development index countries around the world (96). Under the US program, DBS are collected within 24–48 h of birth for >98% of the nearly 4 million infants born each year (97, 98). The DBS are sent to state laboratories to test infants for inborn errors of metabolism and other selected genetic and endocrine disorders. After screening is complete, residual DBS are stored for confirmation of positive results or re-testing as needed. Some states also retain residual DBS for research purposes, although the storage duration varies widely by state, from 30 days to indefinite, and policies regarding DBS retention and use in research continue to rapidly evolve (99).

DBS represent an ideal sample for preleukemic clone screening. They are easily stored, routinely collected for every infant, and, in some states, available for population-based research studies. Despite their many benefits, there are also barriers to using DBS for this purpose, such as variation in storage conditions among different states. For instance, while some states store residual DBS at −20°C, other states store DBS without temperature or humidity control (99). This represents a challenge for RNA-based methods, as RNA can degrade rapidly in DBS, particularly those stored in uncontrolled temperature conditions (100). Additionally, while UCB provides ample input material for many applications, DBS provide a limited quantity of cells. Each Guthrie card spot is ~6 to 10 mm in diameter and may be unevenly filled with blood (101). When a spot is completely filled to its borders, it contains approximately 50 μL of whole blood, and the amount of DNA or RNA that can be extracted will vary according to storage conditions and duration (102, 103). Despite these limitations, the widespread availability and ease of collection and storage of DBS make it an appealing target for new method development and eventual translational application. Additionally, recent studies have demonstrated that archived DBS are suitable for some RNA-based applications, including gene expression profiling (104–106).

#### Novel Detection Methods

As technology evolves, it may enable novel leukemia translocation detection methods. Detection of rare translocations can be achieved using DNA if the breakpoint region is small, by placing PCR primers in both translocation partner genes to create a translocation-specific PCR product. However, the breakpoints for most translocations, including the most common *ETV6*/*RUNX1*, occur within a large region of the genome (described in *Translocation Prevalence Estimates and Variation* section), and this has driven the use of RNA-based methods. Next-generation sequencing (NGS) is under-explored for the detection of translocations in newborn samples. Utilization of an NGS approach will require enrichment of target sequence. For instance, to detect *ETV6*/*RUNX1* would require enrichment of *RUNX1*-specific cDNA using sequence specific oligos that are placed in exons 2, 3, and 4 in a reverse transcription assay (107). This procedure would create a pool of cDNA containing both wild-type *RUNX1* and *ETV6*/*RUNX1* fusion transcripts. The entire pool of cDNA could be used for library creation using Nextera XT transposase, which is optimal for use with low DNA inputs. However, an NGS approach has many challenges, including low quality or degradation of the input RNA, insufficient gene-specific cDNA, the need for high sequencing depths, and high cost, which limit feasibility for large-scale implementation. Additionally, this approach would require extensive bioinformatic resources.

Another possible innovative approach includes the application of digital PCR (dPCR), which has recently been utilized in a backtracking study of childhood ALL (55). dPCR is an attractive target for translocation screening given its sensitivity and specificity and the exceptional performance of dPCR for low copy number events. However, this approach requires careful design of primers and probes to enable translocation detection across multiple breakpoints and would require determination of a positive droplet threshold to determine true-positive samples. The innovative GIPFEL procedure, which utilizes DNA and inverse PCR, overcomes both the RNA quality/quantity problem and the “large breakpoint span” problem; it is an exciting development in the detection of translocations in the research setting.

### Epidemiologic Studies Enabled by New Methods of Screening

The development of robust translocation detection methods utilizing DBS would open several opportunities for future epidemiologic and natural history studies of childhood leukemia. First, large studies of the prevalence of translocations in the general population of newborns could quickly determine whether the frequency of mutations varies in conjunction with known risk factors for overt ALL. Second, these methods would enable establishment of the first prospective epidemiologic studies of ALL risk by concentrating on children with translocations present at birth to determine the early life factors that promote or arrest their expansion. With serial sampling, researchers could also examine translocation persistence rather than overt leukemia as a study outcome. Should these studies suggest an intervention, they could motivate initiation of newborn screening and prevention of childhood leukemia. Success of these studies depends on a robust assay that captures translocation status in newborn DBS, even those not stored in ideal conditions.

### Newborn Screening for Childhood Leukemia

Although there is evidence that some leukemic translocations, such as *ETV6*/*RUNX1*, occur at substantially higher rates *in utero* than the rate of overt leukemia (70), there may be translocations whose frequency of *in utero* initiation is near that of the specific leukemia subtype to which they give rise. For instance, although the studies examining the prevalence of *KMT2A*/*AFF1* in healthy newborns were limited in sample size (Table 2), they suggest that this translocation occurs infrequently in the population of unselected newborns. If the incidence of the *KMT2A* rearrangements is close to that of infant leukemia, they may be nearly deterministic for infant leukemia development and newborn screening may be warranted. Indeed, infant leukemia is an attractive target for newborn screening given that it is a serious disease with onset in the 1st year of life for which there are available treatments. Despite the potential benefits of early detection and intervention for infant leukemia, a newborn screening program for infant leukemia presents several challenges. First, depending on the method of fusion detection, the addition of screening for preleukemic clones may be cost prohibitive on a population-wide basis. Additionally, a positive result would likely provoke a considerable amount of parental anxiety related to the newborn's well-being. Finally, infant leukemia patients experience high rates of glucocorticoid resistance (108), relapse, and emergence of chemoresistant cell populations (109), particularly among *KMT2A*-rearranged ALL. Therefore, the clinical utility of early intervention enabled by newborn screening is unclear for this population and requires additional research.

## Conclusion

More than 70% of infant leukemia cases and >40% of ALL cases diagnosed age 1–9 years contain cytogenetic profiles that have been found to occur *in utero* through backtracking and twin studies and have been positively identified in newborn blood spots. This includes *KMT2A* rearrangements, common in infant leukemia, which confer very poor survival, and both *ETV6*/*RUNX1* and hyperdiploidy, which represent the majority of ALL diagnosed at ages 1 to 9 years.

Resolving the uncertainty regarding the prevalence of translocation at birth is an important public health issue with potential implications for newborn screening. However, previous studies among known leukemia cases require determination of the exact translocation breakpoint from diagnostic samples, while methods used in previous studies of healthy newborns are cumbersome and require the use of UCB. Developing a robust method using newborn DBS that can identify rare clones without false positives will have high value for future research efforts into the etiology of leukemia.

## Author Contributions

ELM and LGS: article concept and design. ELM: drafting of the manuscript and statistical analysis. All authors: critical revisions of the manuscript for important intellectual content. All authors listed have made a substantial, direct and intellectual contribution to the work, and approved it for publication.

## Conflict of Interest

The authors declare that the research was conducted in the absence of any commercial or financial relationships that could be construed as a potential conflict of interest.
